# Lipofuscin-associated photo-oxidative stress during fundus autofluorescence imaging

**DOI:** 10.1371/journal.pone.0172635

**Published:** 2017-02-24

**Authors:** Michel M. Teussink, Stanley Lambertus, Frits F. de Mul, Malgorzata B. Rozanowska, Carel B. Hoyng, B. Jeroen Klevering, Thomas Theelen

**Affiliations:** 1 Department of Ophthalmology, Radboud University Medical Center, Nijmegen, the Netherlands; 2 Department of Applied Physics, University of Twente, Enschede, the Netherlands; 3 School of Optometry and Vision Sciences, Cardiff University, Wales, United Kingdom; University of Florida, UNITED STATES

## Abstract

**Purpose:**

Current standards and guidelines aimed at preventing retinal phototoxicity during intentional exposures do not specifically evaluate the contribution of endogenous photosensitizers. However, certain retinal diseases are characterized by abnormal accumulations of potential photosensitizers such as lipofuscin bisretinoids in the retinal pigment epithelium (RPE). We sought to determine these contributions by a numerical assessment of in-vivo photo-oxidative stress during irradiation of RPE lipofuscin.

**Methods:**

Based on the literature, we calculated the retinal exposure levels, optical filtering of incident radiation by the ocular lens, media, photoreceptors, and RPE melanin, light absorption by lipofuscin, and photochemical effects in the RPE in two situations: exposure to short-wavelength (*λ* = 488 nm) fundus autofluorescence (SW-AF) excitation light and exposure to indirect (diffuse) sunlight.

**Results:**

In healthy persons at age 20, 40, and 60, respectively, the rate of oxygen photoconsumption by lipofuscin increases by 1.3, 1.7, and 2.4 fold during SW-AF-imaging as compared to diffuse sunlight. In patients with STGD1 below the age of 30, this rate was 3.3-fold higher compared to age-matched controls during either sunlight or SW-AF imaging.

**Conclusions:**

Our results suggest that the RPE of patients with STGD1 is generally at increased risk of photo-oxidative stress, while exposure during SW-AF-imaging amplifies this risk. These theoretical results have not yet been verified with in-vivo data due to a lack of sufficiently sensitive in-vivo measurement techniques.

## Introduction

Fundus autofluorescence (AF) imaging visualizes the accumulation of fluorophores that constitute a substantial fraction of lipofuscin in the retinal pigment epithelium (RPE [1]). The pigments of lipofuscin are produced in the membranes of photoreceptor outer segments from non-enzymatic reactions of vitamin A aldehyde [2–5]. This fluorescent material is transferred to RPE cells within phagocytosed outer segment disks [6, 7], and becomes deposited in the lysosomal compartment of the cells. As a result, RPE-lipofuscin accumulates with age [8] and fundus AF increases linearly with age although subjects vary in terms of intensities [9]. Short-wavelength AF (SW-AF, *λ*_exc_ = 488 nm, unless stated otherwise) is commonly regarded as a way to monitor the status of RPE cells, with areas of high AF indicating increased lipofuscin levels and areas of absent AF indicating loss of RPE cells.

The retinal radiant exposure of the SW-AF excitation light is far below ANSI safety thresholds [10]: SW-AF-imaging with the widely used Spectralis device is safe for up to 8 hours (9 J·cm^-2^), whereas typical examinations irradiate the retina for less than 5 minutes (<0.1 J·cm^-2^). These thresholds were based on cross-sectional data of the effects of light on a cellular level, designed to protect the eye and skin from accidental light exposure. To reduce ocular exposures, the International Commission of Non-Ionizing Radiation Protection has also provided guidelines for ophthalmic instruments [11]. Commercial or experimental ophthalmic instruments adhere to these standards with additional constraints for intentional exposures [12], and may thus be considered safe with regard to short-term effects.

However, the ANSI thresholds and Commission guidelines do not specifically evaluate the contribution of endogenous photosensitizers in enhancing a patient’s susceptibility to retinal phototoxicity. In fact, patients with certain retinal diseases may be highly susceptible to phototoxicity [13, 14]. This has led to concerns that patients with recessive Stargardt disease (STGD1) may be at risk for light toxicity during SW-AF imaging [15]. In patients with STGD1, photochemical damage may involve changes in molecules within the visual cycle such as all-trans-retinal [14, 16]. In fact, removal of all-trans-retinal from the photoreceptor outer segment disks is impaired [17], which leads to an accelerated accumulation of lipofuscin bisretinoids in the RPE. Some of these bisretinoids have been identified as potent photosensitizers in animal studies. In *Abca4*^*-/-*^ mice, very high intensities (50 mW/cm^2^) of blue light (*λ* = 430 ± 20 nm) irradiation for 30 min caused severe atrophy of photoreceptors and RPE cells with elevated lipofuscin, which was less pronounced in age-matched wild type controls [18]. Conversely, there was no photoreceptor atrophy in *Rpe65*^*rd12*^ mice without RPE-lipofuscin [18]. Whether the mechanism of photochemical damage involves changes in either lipofuscin or molecules within the visual cycle such as all-trans-retinal, patients with STGD1 will be highly susceptible to photic injury [14, 16]. Consistent with this notion, even chronic exposure to normal daylight appears to increase the progression of RPE damage in STGD1 [19]. Evidence from studies with *Abca4*^-/-^ mice indicates that an accelerated accumulation of lipofuscin bisretinoids in the RPE mainly underlies increases in photosensitivity in STGD1 [18]. Whether the lipofuscin in STGD1 shows age-related increases in photoreactivity—as was found in healthy individuals [20]—remains to be determined, since an earlier study [21] examined the RPE of a 24-year old patient and found an ‘abnormal form of lipofuscin’; this material may be more photoreactive than its age would suggest.

We aimed to determine the extent to which the endogenous photosensitizer lipofuscin makes humans more susceptible to photic injury, for which there is lack of empirical evidence. Extrapolation from results from animal studies to humans is difficult because of their considerable differences in light susceptibility. Therefore, we numerically simulated in-vivo photo-oxidative stress in the human RPE subsequent to irradiation of endogenous RPE-lipofuscin, allowing us to estimate this extent. More precisely, we simulated exposure during either SW-AF imaging in common clinical practice or diffuse sunlight, in healthy individuals of different ages and in patients with STGD1. Daylight exposure is not known to cause retinal injury to healthy people except for unintentional and excessive exposures [22], which thus can provide a reference frame of normally harmless effects. Such an approach may yield considerable insight, because it facilitates the identification of gaps in our knowledge of all aspects involved in retinal photo-oxidative stress.

## Materials and methods

### Retinal exposures

#### Exposure to daylight

**Fig 1:**
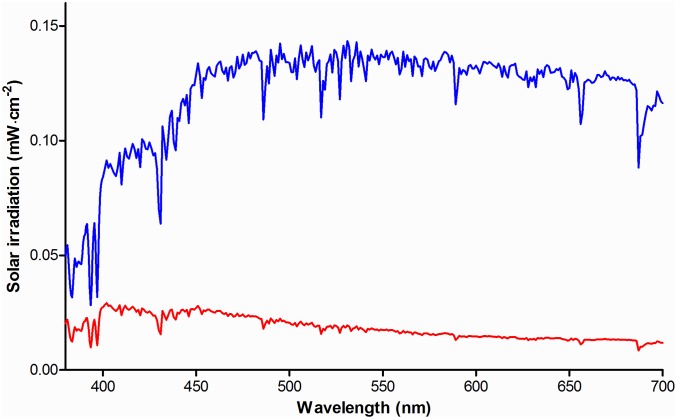
Solar irradiance spectra. Visible range of the ASTM G173-03 solar irradiance spectrum, measured at a global tilt of 37° pointing to the sun (blue). The solar irradiance spectrum when not staring directly at the sun, including light scattered by the atmosphere and light reflected off the earth’s surface, is also shown (red).

We used the solar spectrum of the American Society of Testing and Materials (ASTM G173-03) as a reference for terrestrial solar irradiation [23]. It was measured under atmospheric conditions considered a reasonable average over a period of one year, and pointing to the sun at an inclination of 37°. This inclination corresponds to the approximate average latitude of the 48 contiguous states of the USA. This spectrum includes light scattered by the atmosphere and light reflected off the earth’s surface (Fig 1). In such a scenario of free or Newtonian illumination [24] a distant light source—the sun—irradiates an area *A* larger than the pupil of the eye. The retinal radiant exposure *H*_*r*_ (J·cm^-2^) can then be expressed as a function of corneal radiant exposure *H*_*c*_ (J·cm^-2^ [12]).

$$H_{r}=H_{c}\frac{A_{\text{pupil}}}{A_{\text{retina}}}\approx H_{c}\tau \frac{d_{p}^{2}}{f_{e}^{2}\alpha^{2}}=L_{s}\tau \frac{\pi }{4}{\left(\frac{d_{p}}{f_{e}}\right)}^{2},$$ (1)

with the pupil diameter (*d*_*p*_), the eye’s focal length (*f*_*e*_), the visual angle of the source (*α*), and the ocular media transmission (*τ*). For free illumination, the retinal radiant exposure *H*_*r*_ can also be expressed as a function of the radiance of the source (*L*_*s*_, unit J·sr^-1^), independent of *α* (Eq (1), third term [12]). Using normative data of the pupil diameter at different ages measured under various lighting conditions [25], we calculated an average pupil diameter *d*_*p*_ at age 20, 40, and 60 of 3.8, 3.5, and 3.2 mm, respectively (Section A in S1 Text). We assumed that the pupil is adapted to daylight luminance without pharmacological dilation and we used an average focal length *f*_*e*_ of the eye of 17 mm.

Because a person will usually not stare directly into the sun, the referenced solar spectrum is an overestimation of the actual solar irradiation entering the eye. We therefore subtracted the ‘direct and circumsolar’ spectrum that measures a 2.5° circle around the solar disk from the aforementioned (‘global tilt’) solar spectrum as an indication of indirect solar irradiation, *i*.*e*., diffuse insolation (Fig 1). To determine the irradiance *L*_*s*_ (W·cm^-2^·sr^-1^) of this diffuse scattered light, we used the solid angle Ω of radiation specified for the ASTM reference spectrum, which equals that of diffuse light scattered in a full hemisphere (Ω = 2*π* steradian [23]). Consequently, *L*_*s*_ ≈ *H*_*c*_/2*π*.

To account for absorption in the ocular media, we employed an algorithm that predicts the average media optical density at a given age and wavelength [26, 27]. The algorithm of Van de Kraats and Van Norren is based on six optical density components with the optical density *D*_*λ*_ depending only on wavelength and age [27]. We obtain:

$$H_{r}=\frac{H_{c}}{2\pi }\text{exp}(-D_{\lambda })\frac{\pi }{4}{\left(\frac{d_{p}}{f_{e}}\right)}^{2}.$$ (2)

We used Eq (2) to predict retinal exposures to diffuse insolation at age 20, 40, and 60.

#### Autofluorescence imaging

In SW-AF by confocal scanning-laser ophthalmoscopy, the imaging beam enters the eye with a known angle *α* through an entrance pupil smaller than the pharmacologically dilated pupil. In this scenario of Maxwellian illumination, the retinal radiant exposure is the power entering the pupil Φ, divided by the retinal exposed area [12]:

$$H_{r}=\Phi \tau \frac{4}{\pi {\left(f_{e}\alpha \right)}^{2}}.$$ (3)

The blue autofluorescence imaging mode of the widely used Spectralis HRA+OCT employs an optically pumped solid-state continuous wave laser with a wavelength of 488 ± 2 nm and a recommended maximum optical power of 260 μW to excite lipofuscin fluorophores in the fundus. Emitted fluorescence in the wavelength range of 500–680 nm is detected after passing through a barrier filter. We assumed that during AF imaging in a clinical setting, the retina is scanned at the high-speed mode (768 x 768 pixels; 8.9 frames·s^-1^) in square 30° fields. Imaging of 55° fields is performed frequently, although the resulting average retinal exposure will be lower, and it therefore should be safer. Although the Spectralis also offers the possibility of imaging at the ‘high-resolution’ mode with a doubled sampling rate (i.e. each imaged area is effectively probed twice by the imaging beam), the average retinal exposure will remain the same since the beam power, imaging speed, and size of the imaged area remain equal. Under our assumptions, the average retinal radiant exposure in perfectly transparent media is 328 μW·cm^-2^ [28]. Taking media absorption in a healthy 20-year old person [27] into account, it is 190.4 μW·cm^-2^.

### Optical screening in the fundus

#### Photoreceptors

The absorption of light in the neural retina is orders of magnitude lower than that in the RPE [29]. Since our study is focused on the paramacula (about 10° retinal eccentricity), we neglect the influence of macular pigments. Visual pigments in the photoreceptors, however, may contribute to the absorption of light. Therefore, we estimated the visual pigment optical density versus wavelength during daylight exposure.

A luminance of 2.9 photopic cd·m^-2^ is already sufficient to saturate rod electroretinographic responses [30], and therefore the unbleached fraction of rod VP at an illuminance of 4400 cd·m^-2^ (Section A in S1 Text) will be very low—we consequently neglect absorption by rods during either daylight or SW-AF imaging. We used data on the normalized wavelength-dependent optical density of visual pigments in photoreceptor outer segments, measured by microspectrophotometry on ex-vivo human samples [31]. We fitted these data with polynomial functions to obtain the optical density of the entire 380–700 nm wavelength range. These normalized data, expressed in normalized optical density per micron outer segment length, were multiplied by the mean dark-adapted double-pass optical density of each of the photoreceptor types, divided by two to obtain single-pass optical density. These numbers were multiplied by the mean length of photoreceptors at 10° retinal eccentricity [32]. Next, we multiplied the result by the retinal area fraction occupied by cone photoreceptors at 9.2° retinal eccentricity, as derived from electron microscopy data obtained by Curcio et al. (1990 [33]), and by the relative numbers of the different cone types as published by Dartnall et al. (1983 [31]). Finally, we used data on the steady-state bleach fraction of visual pigments at an illuminance of 4400 cd·m^-2^ to derive the fraction of unbleached visual pigments. This fraction was determined to be 0.08, which we multiplied with the wavelength-dependent optical density of cones.

#### Melanin in the retinal pigment epithelium

The flux of photons impinging on lipofuscin is reduced due to optical screening by melanin granules situated apically in RPE cells. RPE melanin consists largely of eumelanin [34], which is able to dissipate approximately 90% of incident UV energy as heat [35]. We performed a Monte-Carlo (MC) simulation of light scattering and absorption by melanin in the RPE to investigate optical screening by melanin in healthy people of different ages and in patients with STGD1. MC methods are a standard approach in numerical simulation and the basic methodology in simulating scattering of light in human tissues is, by now, strongly established. MC methods have been employed with great success in order to predict the properties of light scattering in human tissues [36–39]. Our calculation of light scattering by melanosomes was similar to an earlier study by Cracknell et al. (2007), who used MC methods to investigate iris melanosomes [40]. Our calculation of light absorption by melanin was different from the calculation by Cracknell et al.: we based it on empirical data of the absorption spectrum of melanin. Details of this MC simulation of in-vivo optical attenuation by RPE-melanin in the paramacular RPE-cells are depicted in Section B of S1 Text. We modeled the paramacular RPE as a single 9 μm thick sheet [41], and RPE-melanin could occupy the apical ≤ 33% of the RPE-cell (inward positive, i.e., the optical path length *l*_melanin_ ranged from 0 to +3 μm). An infinitely thin and non-divergent beam of light (‘pencil beam’) injected 5·10^5^ photons into the system. We varied the thickness of the layer in which the scatterers (melanosomes) are present with age and/or the presence of STGD1, as specified in Section B in S1 Text.

MC-simulations of optical screening by RPE-melanin were performed for specified wavelengths (*λ* = 380, 405, …., 705) for each of four different scenarios: healthy 20-, 40-, and 60- year old paramacular RPE, and 20-year old non-atrophic RPE of a patient with STGD1. MontCarl counted the number of photons that were either absorbed, backscattered (upon refractive passage at the interface between two layers and directed towards negative depth values), or transmitted (the inverse of backscattering; this could therefore include non-scattered and forward scattered photons). From these fractions and the total number of incident photons, we calculated attenuation coefficients (*μ*_*a*, melanin_ and *μ′*_*s*, melanin_) and the optical density (OD_melanin_) as:

$$\mu_{\text{a, melanin}}=-\text{log}\left(1-\frac{\left[\text{Absorbed photons}\right]}{\left[\text{Total injected photons}\right]}\right)/l_{\text{melanin}},$$ (4)

$$\mu \prime_{\text{s, melanin}}=-\text{log}\left(1-\frac{\left[\text{Backscattered photons}\right]}{\left[\text{Total injected photons}\right]}\right)/l_{\text{melanin}}\text{,}$$ (5)

and

$$\text{O}{\text{D}}_{\text{melanin}}=\left(\mu_{\text{a, melanin}}+\mu \prime_{\text{s, melanin}}\right)\cdot l_{\text{melanin}}.$$ (6)

### Optical absorption by lipofuscin

The high optical density of each lipofuscin granule may give rise to significant internal optical screening [42]. Granules in the basal part of the cell may therefore receive little or no light; resulting in a poor correlation between the RPE-lipofuscin concentration and total light absorbed. This may explain why—at present—we have no evidence that lipofuscin photo-oxidation varies with the lipofuscin bisretinoid concentration.

Calibrated SW-AF measurements have shown that patients with STGD1 exhibit substantially increased fluorescence from RPE-lipofuscin [43–45]. This may be ascribed to either increased fluorescence efficiency of lipofuscin bisretinoids, increased absorption of excitation energy, or both. The ‘dark’ or ‘silent’ choroid sign on fluorescein angiography, present in 37–50% of patients [46, 47], indicates a considerable reduction in light transmission (*λ* = 488 nm) through the RPE [46, 48, 49]. Increased backscatter and/or absorption from lipofuscin may underlie this phenomenon; however, increased backscatter is highly unlikely to be the sole cause. Finally, mouse studies have shown that the amount of the lipofuscin bisretinoid A2E decreases in-vivo when retinal light exposure increases, due to lipofuscin oxidation and subsequent degradation [50].

We incorporated light absorption by lipofuscin into our simulation because of these indications. Although an accurate approximation of the fraction of light absorbed could be obtained with an MC simulation, as far as we know there are no empirical data on certain optical parameters of lipofuscin granules. These parameters include the granule size distribution, wavelength-dependent absorption- and scattering cross-sections, and empirical data on the angular scattering function. We therefore took a different approach, based on the principle that light absorption tends to correlate with the granule concentration (*n*_*g*_) and the optical path length (*l*) through these granules. Hence, we considered their product (*n*_*g*_ · *l*) indicative of light absorption.

Although electron microscopy of the RPE of patients with STGD1 shows massive accumulations of lipofuscin in the posterior pole [21], it is difficult to obtain an exact value of (*n*_*g*_ · *l*) based on these images. In mice, however, the concentration of a major fluorophore of lipofuscin (A2E [51]) was found to correlate with the calibrated fluorescence intensity from RPE-cells [52]. Since similar data [28] are available both in healthy people [44] and patients with STGD1 [45], estimations of (*n*_*g*_ · *l*) in STGD1 based on fundus AF would be an alternative. We tested the feasibility of such estimations by determining the correlation between (*n*_*g*_ · *l*) and SW-AF intensity (detailed in Section C [a] of S1 Text). The individual of whom a SW-AF image is depicted has given written informed consent (as outlined in the PLOS consent form) to publish this image.

### Oxygen photoconsumption by lipofuscin granules

The goal of our simulation was to compute oxygen uptake by lipofuscin granules in-vivo under the considered exposure regimes, because oxygen photoconsumption by lipofuscin can serve as an indicator of lipofuscin oxidation [20]. We considered in-vitro oxygen uptake measurements on isolated human RPE lipofuscin granules by Rozanowska et al. (2004) [20] to be—at present—the most appropriate basis for this calculation. Firstly, their measurement setup and results were described in sufficient detail to allow for meaningful and quantitative comparisons with in-vivo exposure conditions. Second, isolated—but intact—human RPE lipofuscin granules of different ages were used, and the pH of the medium is comparable to that in-vivo. As such, these two factors are representative of physiological conditions. The results obtained in sections 3.1 and 3.2 allow us to estimate the flux of photons impinging on RPE lipofuscin granules in-vivo, and studies on photosensitizers have shown a strong relationship between total light absorbed and oxygen uptake [53, 54]. Therefore, the results obtained in sections 3.1 to 3.3 can be regarded as variables influencing oxygen uptake by lipofuscin, and are applicable to an in-vivo milieu. We determined the corresponding values of these variables applicable to the in-vitro measurements by Rozanowska et al. (2004) [20]. By normalizing for differences in these variables in-vivo, we predicted the rates of oxygen uptake, were they measured in-vivo in the RPE. Details on this normalization are described in Section D of S1 Text.

## Results

### Retinal exposures

**Fig 2:**
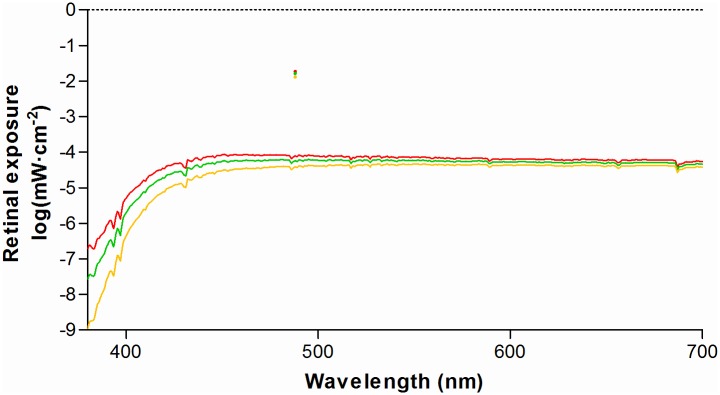
Retinal exposure from diffuse solar irradiation compared to excitation light of short-wavelength retinal auto-fluorescence. At *λ* = 488 nm the peak height is indicated by single colored dots. Exposures were calculated by Eqs (2) and (3), respectively. Exposures in ocular media of different ages are plotted; 20 year-old (red), 40 year-old (green), and 60 year-old (orange).

The retinal exposure (mW·cm-^2^) during daylight or typical SW-AF imaging sessions was corrected for absorption and scattering in the lens and media (plotted in Fig 2).

### Optical screening in the fundus

#### Photoreceptors

**Fig 3:**
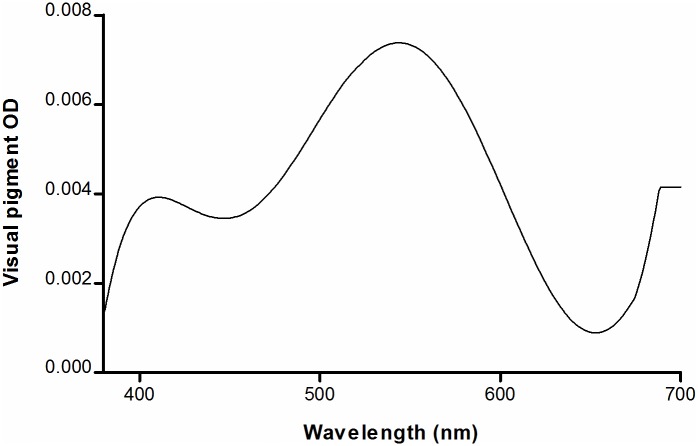
Optical screening by visual pigments (VP) in the outer segments of paramacular photoreceptors. The wavelength-dependent single-pass optical density (OD) of light passing through the outer segments was calculated under conditions of daylight illuminance, amounting to 4400 photopic cd·m^-2^. See text for details.

The calculated visual pigment optical densities during daylight exposure, at a luminance of 4400 photopic cd·m^-2^, are shown in Fig 3. Under this condition, the optical screening by photoreceptors in the paramacula ranges between 0.002 to 0.008 optical density units (0.46–1.83%), and is therefore negligible.

#### Melanin in the retinal pigment epithelium

**Fig 4:**
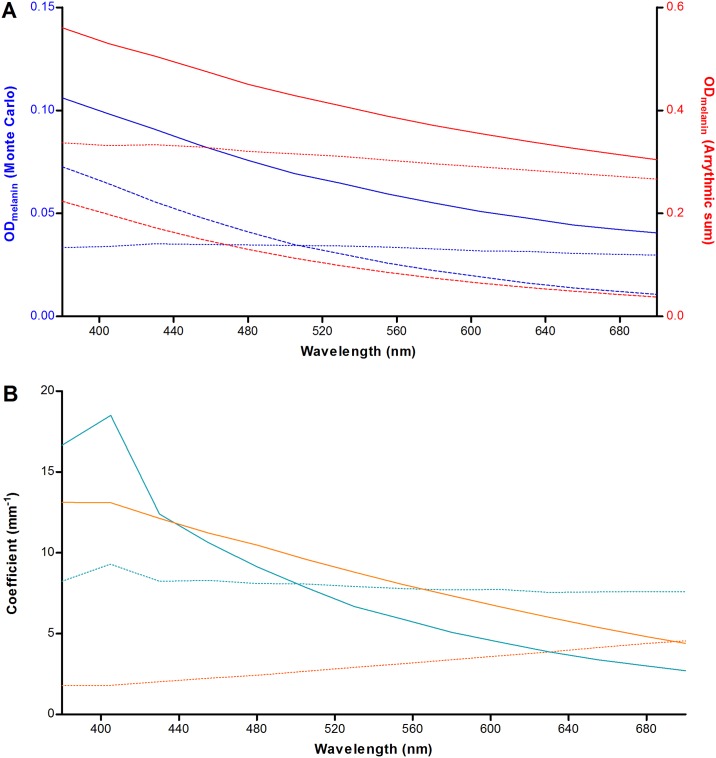
Relative contributions of light scattering and absorption by RPE-melanin. (A) Agreement between Monte-Carlo simulation and theory, plotted based on conditions in the paramacular RPE of a healthy 20-year old person. The optical density (OD) was calculated based on the product of the attenuation coefficient and the melanosome layer thickness (*l*_melanin_). Attenuation by absorption (striped line), scattering (dotted line), and total attenuation (straight line) are plotted separately. The MC results are shown in blue (left Y-axis) and the theoretical result is shown in red (right Y-axis). See text for details. (B) Simulations of a thin (3 μm; blue) and thick (52.5 μm; orange) layer of melanosomes. In case of thicker layers, there is a dominance of the absorption coefficient (*μ*_*a*, melanin_, straight lines) over the backscattering coefficient (*μ′*_*s*, melanin_, striped lines) for all tested wavelengths.

The MC results are plotted in Fig 4. It can be seen that absorption dominates over scattering at *λ* < 505 nm. We evaluated whether this phenomenon is caused by wavelength-dependent differences in the absorption and scattering properties of the melanosomes. By taking the product of each granule class’ concentration and absorption/ scattering cross-section, and taking the arrhythmic sum of all granule classes in the medium, the theoretical absorption coefficient (*μ*_*a*, melanin_) and scattering coefficient (*μ*_*s*, melanin_) can be determined. The reduced scattering (backscattering) coefficient can be calculated by including the scattering anisotropy factor (*g*). It varies from -1 for complete backscattering, through 0 for isotropic scattering, to +1 for complete forward scattering, We calculated the backscattering coefficient by *μ′*_s, melanin_ = *μ*_s, melanin_ (1 − *g*). An estimate of light attenuation due to absorption (OD_a, melanin_ = *μ*_a, melanin_ · *l*_melanin_), backscattering (OD_s, melanin_ = *μ′*_s, melanin_ · *l*_melanin_), and the total optical density (Eq 6) can then be made. As can be seen in Fig 4A, scattering is expected to dominate over absorption for all wavelengths under investigation, which is in contrast to the MC simulation results. In addition, the MC results show several fold lower attenuation for both scattering and absorption.

**Fig 5:**
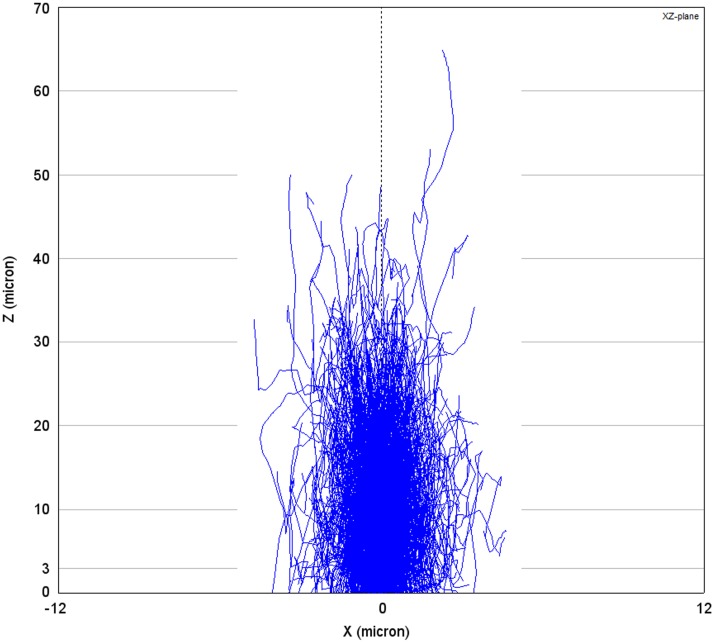
Monte-Carlo simulation of light scattering and absorption in a thick layer of melanosomes. In this plot generated by MontCarl, the optical paths (blue lines) of 3000 photons are ray-traced through a relatively thick layer of RPE-melanosomes at the concentration in-vivo. Photons are injected by an infinitely thin light beam at X/ Y = 0/ 0. The X- and Z-axes, respectively, indicate the lateral and vertical (depth) location in the sample. Most photons are either absorbed or scattered back at Z = 30 μm. At the assumed maximum in-vivo layer ‘thickness’ of RPE-melanosomes (3 μm), a small proportion of photons are backscattered or absorbed.

We found that both of these phenomena can be explained by two aspects: our simulation was performed for a thin layer (3 μm) in combination with a strong tendency for forward scattering in this layer of melanosomes. In this system, photons will deviate from their path by about 30° on average (cos^−1^ < *g* > = cos^−1^(0.865) = 30.3°) at each scattering event, which indicates that randomization of the direction of scattering occurs only after several scattering events. This would suggest that more backscattering occurs when the melanosome layer is thicker. We tested this suggestion by simulating a melanosome layer of either 3 μm or 52.5 μm with an average transmission of photons of 89.4% and 6.6%, respectively (Fig 4B). We found that, in the case of the thicker sample, absorption actually dominates over scattering for all tested wavelength and an overall reduction in the backscattering coefficient *μ′*_*s*, melanin_. This suggests that, although a photon may only backscatter after a given number of scattering events, it becomes increasingly more likely that the photon will be absorbed before it reaches that point. The effect of simulating a thin sample is also illustrated in Fig 5. Thus, in our MC simulation (3 μm), photons have a greater tendency for absorption as compared to backscattering at shorter wavelengths.

**Fig 6:**
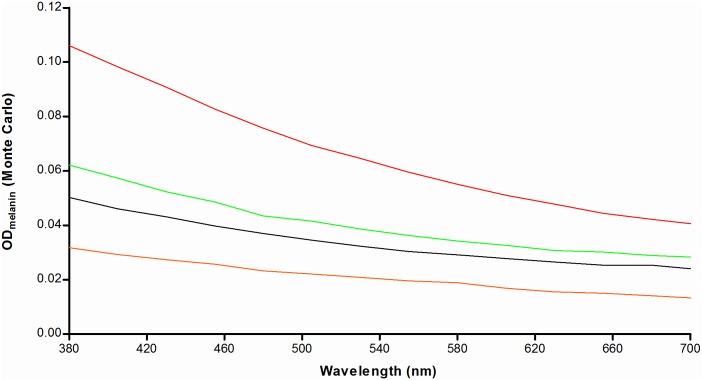
Light attenuation by RPE-melanin in-vivo varies with age and the presence of Stargardt disease. We calculated the total optical density (OD) of paramacular RPE-melanin versus wavelength of incident radiation with Eqs 4–6 based on results of Monte-Carlo simulations. Colored lines indicate attenuation in healthy people of different ages: 20 (red), 40 (green), and 60 (orange). The same is shown for a 20-year old patient with STGD1 (black).

The MC results for the various scenarios tested are shown in Fig 6. We found optical screening by melanin in 20-year old patients with STGD1 to be less than half of that in age-matched controls, with the difference diminishing at longer wavelengths.

### Light absorption by lipofuscin

**Fig 7:**
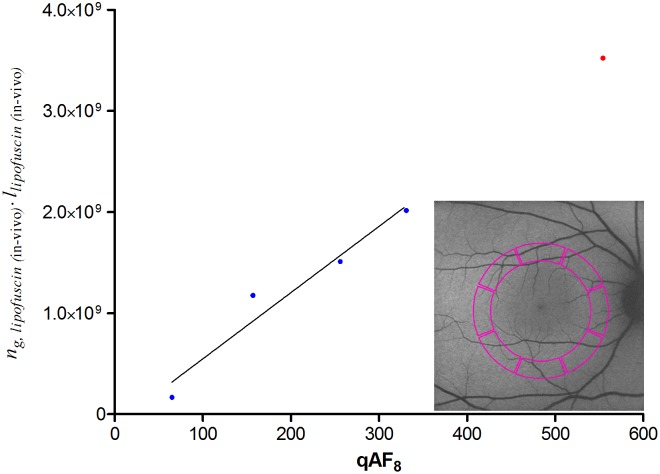
Correlation between calibrated SW-AF measurements and histologic data on lipofuscin granules. We considered the product of optical path length (*l*) and granule concentration (*n*_*g*_) to be indicative of light absorption by lipofuscin granules. Here, we tested whether this product correlates with calibrated SW- AF measurements published earlier (‘qAF_8_’ [28, 44, 45]), possibly allowing an estimation of this product (*n*_*g*_ · *l*) in patients with STGD1 based on their qAF_8_ values. qAF_8_ values were measured in the posterior pole of the fundus (colored area in the inset). Blue dots represent average values of healthy people of different age-ranges; the red dot represents average values of patients with STGD1 (age < 30 years). Pearson’s correlation (r = 0.97) was significant (*P* = 0.0259); therefore, a linear regression analysis was performed with data from healthy people (solid line). With the average qAF_8_ value of patients with STGD1, we extrapolated the value of (*n*_*g*_ · *l*) in STGD1 prior to atrophy of the RPE (red dot).

As shown in Fig 7, we found a strong correlation between calibrated SW-AF measurements (qAF_8_) and values we consider indicative of light absorption by lipofuscin (*n*_*g*_ · *l*). Based on a linear regression model and our calculated average qAF_8_ value of patients with STGD1, we interpolated the value of (*n*_*g*_ · *l*) in these patients.

### Oxygen photoconsumption by lipofuscin

**Fig 8:**
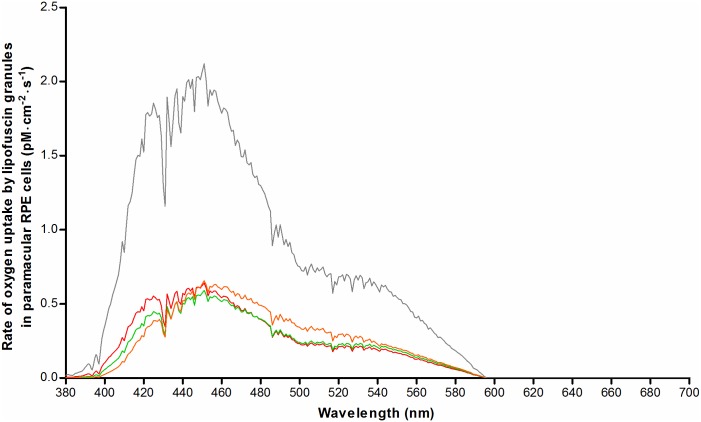
Numerical simulation of oxygen uptake by lipofuscin in paramacular RPE in-vivo during exposure to diffuse sunlight. Oxygen uptake was calculated based on results from a previous investigation of oxygen uptake by isolated human lipofuscin granules [20], after correction for factors affecting retinal exposure levels in-vivo (see text). Results were plotted for healthy people of different ages: 20-year old (red), 40-year old (green), and 60-year old (orange). Results for 20-year old patients with STGD1 are also shown (grey).

**Fig 9:**
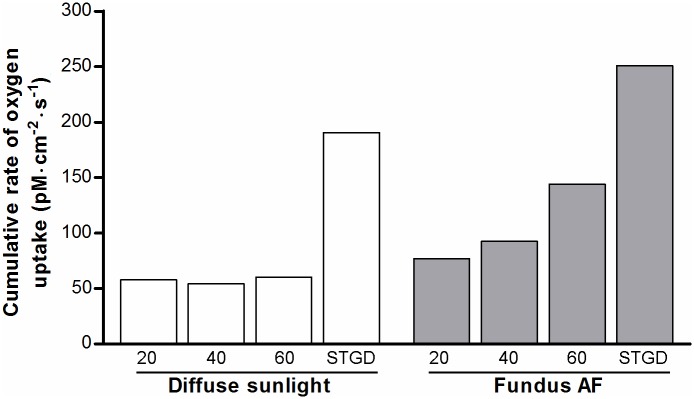
Total rates of oxygen uptake by lipofuscin during light exposure. Rates of oxygen uptake (see Fig 8) were integrated along the wavelength of incident radiation to obtain the total rate of O_2_-uptake, as an indication of cellular oxidative stress in-vivo during exposure to diffuse sunlight (white bars) or during SW-AF imaging (grey bars). X-axes: Age of healthy individuals, or patients with STGD1 (age, 20).

We used the results obtained in sections 3.1–3.3 together with data on the oxygen concentration in the RPE in-vivo to normalize for differences with in-vitro studies on isolated lipofuscin granules (Eq. G in S1 Text) [20]. Fig 8 shows age-related differences in the rate of oxygen uptake (pM·cm^-2^·s^-1^) during sunlight exposure. This is particularly evident for short-wavelength visible light. However, in patients with STGD1, we found an amplification of the rate of oxygen photoconsumption regardless of wavelength. We integrated the results along *λ* to better compare results for different ages, and healthy versus STGD1 (Fig 9). This also facilitates a comparison of low-intensity, broadband radiation (diffuse sunlight) and high-intensity narrowband laser light (SW-AF excitation light). Interestingly, the total rate of oxygen uptake during diffuse sunlight exposure in-vivo varies little with age according to our simulation. During SW-AF, however, oxygen uptake increases considerably with advancing age. The results suggest that oxygen uptake by lipofuscin is increased by about 3.3-fold in 20-year old patients with STGD1 as compared to age-matched controls. To be more specific, during diffuse sunlight and SW-AF imaging, this fold-increase is 3.292 and 3.264, respectively. When comparing oxygen uptake during either exposure to diffuse sunlight or to the SW- AF excitation light, we found a 1.33-, 1.70-, and 2.39- fold increase for healthy individuals aged 20, 40, and 60, respectively. For patients with STGD1, we found a 1.32- fold increase, i.e. close to that in age-matched controls.

## Discussion

Herein, we performed a comprehensive simulation of photo-oxidative stress in the RPE in-vivo, which suggests that lipofuscin granules have a 3-fold higher oxygen uptake and light absorption in patients with STGD1 compared to age-matched controls. To our knowledge, this is the first study to report STGD1 patients’ relative sensitivity to light. We incorporated all known factors influencing light-induced oxygen consumption by RPE lipofuscin, insofar sufficient empirical data was available.

### Optical screening in the fundus

We identified differences in optical attenuation (*μ*_*a*, melanin_) by RPE melanosomes between our simulated in-vivo data and earlier ex-vivo studies. Weiter et al. (1986) [41] found a total attenuation of 0.022 ± 0.008 OD·μm^-1^ in the apical part of RPE-cells (*λ* = 500–600 nm). This agrees well with the pooled average result of our MC simulation for healthy people aged 20–60 (0.020 ± 0.002 OD·μm^-1^). The difference in *μ*_melanin_ may lie in two facts. First Weiter et al. could not distinguish between melanin and melanolipofuscin in their measurements [41]. Our MC simulation would probably have shown a higher *μ*_melanin_ if we had included melanolipofuscin granules, since the latter granules are known to accumulate with advancing age concomitant with reductions in melanosomes [55]. Second, our simulated ‘layer’ of melanosomes (1–3 micron) was thinner than the histologic sections used by Weiter et al. (8 micron) [41]. As we showed in Fig 5, there is a higher proportion of backscattered photons in our simulation as compared to their study, causing an overall higher *μ*_melanin_. Therefore, these two facts taken together might explain the aforementioned slight difference with histologic data, in terms of optical attenuation by melanin in the RPE.

However, optical screening by melanin only marginally protects lipofuscin against irradiation. At an OD_melanin_ of 0.05, only about 11% of the incident light is filtered. Assuming that optical parameters of melanosomes are unchanged in STGD1—as indicated by their normal morphological appearance [21]—our MC simulation showed a 50% lesser screening effect as compared to age-matched controls (Fig 6). Therefore, our data indicates that the apical displacement of melanin in RPE cells of patients with STGD1 [21] is of little consequence with regard to intracellular optical screening.

### Light absorption by lipofuscin

Comparison of light-induced oxidative stress in patients with STGD1 versus healthy controls requires correction for differences in light absorption. Earlier studies used lipofuscin AF as an indication of the concentration of fluorophores [8, 41]. Because calibrated SW-AF measurements [28] are the only quantitative in-vivo indication of the fluorophore concentration, we investigated its correlation with histologic data of the concentration of lipofuscin granules. The fraction of light absorbed (*A*) can be calculated by the formula *A* = *n*_*g*_ · *l*·*σ*_a_ [56], and as shown in Fig 7, we found calibrated SW-AF and (*n*_*g*_ · *l*) to be linearly proportional. We considered the latter directly related to the amount of light absorption, because of two indications of an age-invariant absorption cross-section (*σ*_*a*_). First, our image analysis of previously published [42] electron microscopy images shows no age-related difference in granule size (Section C [b] in S1 Text), ruling out a change in the amount of light scattering. Second, the optical density of lipofuscin granules decreases only slightly (0–14%) with age [42]. In the context of unchanged scattering by these granules, absorption will only marginally change with age. One aspect of note is the increased fluorescence efficiency of oxidized bisretinoids [57], with the oxidized form of A2E being the strongest fluorophore among them [58]. Also, in lipofuscin granules, the ratio of oxidized A2E versus unoxidized A2E increases considerably with age [58]. These results indicate that increased qAF may not correspond with equally increased light absorption. Another aspect to consider is the effect of internal optical screening among lipofuscin granules at high concentrations, due to the high optical density of each granule [42]. This would also cause a lack of linear proportionality between total light absorbed and the granule concentration, especially at high concentrations. Since these two aspects would lead to an overestimation of the amount of light absorbed at high qAF_8_ or high granule concentrations, a linear relationship between qAF_8_ and (*n*_*g*_ · *l*) may be expected. On the other hand, this means that light absorption in patients with STGD1 is probably increased by less than 3-fold.

### Oxygen uptake

Our results suggest that RPE cells of patients with STGD1 are at increased risk of oxidative stress. During SW- AF-imaging, the potential for oxidation almost doubles from age 20 to age 60 (Fig 9). We found a 3.3-fold increase in the rate of oxygen uptake in 20-year old patients with STGD1 relative to that in age-matched controls, regardless of the exposure regime. However, we cannot conclude whether the oxidant/anti-oxidant balance is affected, and if permanent impairment will occur to the RPE or photoreceptor cells. RPE cells are highly resistant to oxidative stress [59], but survival of RPE cells under light stress is largely determined by the relative concentrations of lipofuscin and melanosomes [60]. This balance is clearly less favorable in patients with STGD1; the limited anti-oxidative capacity afforded by melanosomes may prove insufficient to cope with situations of increased oxidative stress when it would normally suffice. In any case, indirect effects of oxidative stress that likely cause damage to photoreceptors have been proven, in terms of a decrease of outer segment phagocytosis by RPE cells [61, 62]. Impairments in this key function of RPE cells can result in retinal degenerations [63].

### Limitations and perspectives

This study had several limitations. First, melanosomes can reduce iron-mediated oxidation in RPE in-vitro by protecting against redox-active metal ion-mediated oxidation [64, 65]. However, whether the net result of this process is anti- or even pro-oxidant depends on many factors, such as relative concentrations of metal ions, small molecular weight iron chelators and melanin-binding sites, presence of oxygen, and irradiation conditions [65]. Due to lack of related in-vivo data on these parameters and their interactions, we omitted this part from our simulation of oxidative stress. Second, the aerobic photoreactivity of melanosomes and melanolipofuscin was not taken into account. These granules display about 6- and 3-fold less oxygen uptake upon irradiation as compared to lipofuscin granules [66], respectively. In addition, compared to lipofuscin, they have a relatively high yield of hydrogen peroxide [66], which has a long half-life [67] and thus is less prone to cause unwanted oxidative damage. Also, melanolipofuscinogenesis involves a gradual fusion of two granule types [68], and optical characteristics and oxygen uptake may change over the course of this process, which may be difficult to model accurately in an MC simulation.

Although we calculated oxygen uptake in STGD1 under the assumption that the photophysical characteristics of RPE lipofuscin remain unchanged, this may prove incorrect considering the ‘abnormal form of lipofuscin’ noted in a histological study of STGD1 [21]. Furthermore, we have not evaluated the consequences of the consumed oxygen; that requires future work that is able to culture RPE cells under replicated in-vivo conditions. We anticipate that this will yield insights into the tolerance of RPE cells to oxidative stress under physiological cell culturing- and light exposure- conditions. It is of interest to note that AF imaging in patients with STGD1 at *λ* = 532 nm, instead of 488 nm, leads to less oxygen uptake at equal optical power: a 0.98-fold versus a 1.32-fold increased uptake relative to daylight, respectively.

## Conclusions

Our numerical simulation of susceptibility to phototoxicity in health and disease indicated a substantial increase in the rate of oxygen uptake by lipofuscin in patients with STGD1. However, sufficient empirical data is lacking on the molecular dynamics of the interplay between increased oxygen uptake, synthesis of oxygen radicals, anti-oxidants, and mechanisms leading to permanent retinal damage. Unfortunately, current in-vivo measurement techniques are insufficiently sensitive to show any effect of sub-threshold light damage in patients. Considerable insight into these dynamics can be gained by numerical simulation and comparisons with empirical data obtained in cells cultured in replicated (patho)physiological conditions. Simulations can also elucidate the relative vulnerability of various retinal areas with different characteristics. We anticipate that this can eventually lead to personalized risk assessments of patients undergoing retinal light exposure in various settings. In high risk patients it may be advisable to largely avoid chronic exposure to light with wavelengths less than 500 nm and to choose autofluorescence excitation above this wavelength.

## Supporting information

S1 Text Detailed methods and calculations. (DOCX)

S1 Data Data used in this study. The data used in this study are seperated per section and follow the same order as listed in the Appendix (S1A–S1D Text). Most sections are further subdivided into separate parts (a, b, c), and for each a descriptive titel is given in the appropriate tab of the spreadsheet.
(XLSX)
