# Supplementary material for: Lipofuscin-associated photo-oxidative stress during fundus autofluorescence imaging
Source: PLoS One. 2017 Feb 24;12(2):e0172635. doi: 10.1371/journal.pone.0172635 (PMC5325292; doi:10.1371/journal.pone.0172635)
Supplement: S1 Text — (DOCX) [file pone.0172635.s001.docx]

S1 Text. Detailed methods

and theoretical considerations.

1. Diameter of pupils adapted to daylight
2. Optical screening by melanin in-vivo
3. Optical absorption by lipofuscin in-vivo
4. Oxygen photoconsumption by lipofuscin
5. Table of incorporated parameters
6. **Diameter of pupils adapted to daylight**

The diameters of pupils adapted to daylight were based on normative data measured at 4400 cd·m^-2^ for the ages 20, 40, and 60 [1]. This outdoor lighting condition was chosen, because an overcast day illuminates by about 2200 cd·m^-2^ while full daylight gives about 22700 cd·m^-2^ for the respective illuminances of 1075 and 10752 cd·sr·m^-2^ [2]. We assumed the angle *θ* subtended by the source (daylight) to amount to 45 degrees (0.79 rad), which gives a solid angle Ω of 0.49 sr based on the formula

1. **Optical screening by melanin in-vivo**

In order to determine the optical transmission through melanosomes in the RPE, we performed a Monte-Carlo simulation of light scattering and absorption based on Mie theory. We used the program MontCarl [3], which simulates photons as wave packets (i.e., ‘photons’). MontCarl allows the user to define properties of 1) the source, 2) the scattering volume with internal layers and objects, 3) scattering and absorptive properties of particles with different sizes, 4) descriptions of their location and concentration, and 5) methods of photon detection [4, 5].

Monte-Carlo simulations require at least 5 basic parameters; 1) wavelength of the radiation and refractive indices of the media and scattering particles, 2) the absorption and scattering cross-sections of the particles, 3) the particle concentration, 4) the particle diameters, and 5) the dimensions of the system in which scattering takes place. We will address the relevant calculations only briefly, because optical screening by melanin is not the main focus of this study.

**B (1). Light absorption by melanin granules**

The absorption coefficient of melanin (*μ_a,_* _melanin_) may be estimated with information on the concentrations of eumelanin and pheomelanin monomers in a tissue, and using the extinction coefficients of Sarna and Schwartz (1988) [6, 7]:

| $\begin{matrix} \varepsilon_{\text{eumelanin}}=\left(2.37\cdot10^{4}\text{ cm}^{-1}\text{ M}^{-1}\right)\cdot e^{-0.0056\lambda}, \\ \varepsilon_{\text{pheomelanin}}=\left(1.01\cdot10^{5}\text{ cm}^{-1}\text{ M}^{-1}\right)\cdot e^{-0.0087\lambda}, \end{matrix}$ | (A) |
| --- | --- |

such that the absorption due to melanin is [7]:

| $\mu_{\text{a}}\text{ due to melanin}=(\varepsilon_{\text{eumelanin}}C_{\text{eumelanin}}+\varepsilon_{\text{pheomelanin}}C_{\text{pheomelanin}})\text{ ln(10).}$ | (B) |
| --- | --- |

We calculated the average diameters of melanin granules using scanning electron microscopy images of isolated RPE melanosomes published earlier (Ref [8], Fig. 5B). Since many of these granules are ellipsoidal rather than spherical, we segmented the granules with ImageJ (version 1.48v; <http://imagej.nih.gov/ij/>; provided in the public domain by the National Institutes of Health, Bethesda, MD, USA), and measured their surface area. We calculated an average sphere-equivalent diameter of 0.786 ± 0.17 μm. Assuming that sphere diameter is normally distributed, we can discretize the distribution by defining 9 classes of granules, i.e. each class has a specific size and concentration. MontCarl is able to calculate scattering characteristics of discrete classes of scatterers based firstly on contributions from various angular scattering functions (e.g., 100% Mie function, 100% Henyey-Greenstein, or other combinations), and second with the value of the relevant input parameters of each contribution.

At young age, melanosomes occupy approximately 33% of the apical RPE cell cytoplasm in-vivo [9]. Feeney-Burns determined that melanosomes constitute 8% of the cross-sectional area of an RPE cell in the first two decades of life [10] and, according to the Delesse principle, the area fraction of a tissue component gives an estimate of its volume fraction [11]. From these data we can calculate that each paramacular RPE cell contain about 552 melanosomes, giving a concentration of 9.41·10^11^ granules·ml^-1^. Although RPE melanosomes consist *largely* of eumelanin [12, 13], we made the simplifying assumption that they consist *entirely* of eumelanin monomers. At an average granule diameter of 0.786 μm, the concentration of eumelanin monomers in the paramacular RPE (M) is then 0.111 M, which allows us to calculate (*μ_a,_* _melanin_ [Eq. A]).

At ages 40 and 60, respectively, melanin constitutes 6% and 3.5% of the RPE-cell area/ volume [10]. This decline is coincident with a proportional increase in melanolipofuscin [10] presumably due to gradual fusion of melanosome with lipofuscin granules [14]. This is consistent with observations that 1) the polarized distribution of apical melanin and basal lipofuscin is lost with advancing age [14, 15]; 2) that by age 50 most melanin is incorporated into melanolipofuscin [15]; and 3) that by age 90 melanosomes represent almost all RPE pigment granules [16]. The physical implication of this decline is that the optical path length through melanin decreases with age. According to this principle, melanosomes occupy the apical 25% at age 40 and 14.57% at age 60 while maintaining the same local concentration. Histopathology of a 24-year old patient with Stargardt disease revealed a striking apical displacement of melanosomes to the estimated apical fifth part of peripheral RPE cells [17]. In the posterior pole, a less prominent division line was observed, and melanin content appeared reduced in these cells [17]. We consider it likely that melanolipofuscinogenesis underlies these observations, which may be more complete in posterior RPE-cells as compared to peripheral RPE-cells. Our study is focused on paramacular RPE cells. Therefore, based on these observations we estimate that melanin is concentrated in the apical 1/5^th^ of the paramacular RPE cell and that the basal 50% of these granules have been partially incorporated into melanolipofuscin and no longer contributes to effective optical screening. The layer thickness of ‘pure’ melanin is then approximately 1 micron whereas the local melanosome concentration is increased by 40% higher than normal. The melanosome morphology appeared normal in Stargardt disease [17], and therefore we assume that granule absorption and scattering characteristics remain unchanged.

With *μ_a_* and the granule concentration (*n_g_*) known, the average absorption cross-section of the melanin granules *σ*_a, melanin_ can be calculated with the well-known relation:

| $\sigma_{\text{a}}=\mu_{\text{a}}/n_{g}.$ | (C) |
| --- | --- |

*σ*_a_ is related to the particle diameter *D* and the absorption efficiency (Q_abs_) as:

| $\sigma_{\text{a}}=\frac{\pi D^{2}Q_{\text{abs}}}{4}.$ | (D) |
| --- | --- |

With the wavelength-dependent Q_abs_ known, we were able to calculate the wavelength-dependent absorption cross-section of the melanin granules.

**B (2). Scattering by melanin granules**

Mie theory calculates the angle-dependent scattering function of incident radiation based only on the particle aspect ratio (*X*), the wavelength of incident radiation and the refractive index of the particle material; the former 2 parameters should incorporate the medium of refractive index (*n*). The ability of the ‘full’ Mie function to accurately describe a particle’s angular scattering functions depends on the accuracy of aforementioned input parameters. In contrast, several earlier studies on biological tissues have employed the Henyey-Greenstein (HG) approximation to describe angular scattering [13, 18, 19], and have found it to be appropriate.

As input, the HG-function only requires an accurate estimation of the scattering anisotropy factor (*g*). This parameter can be calculated by employing Mie scattering theory [20, 21], and using knowledge of the particle aspect ratio and the relative complex refractive index $m=(n+in')/N$ of the material. The interested reader is referred to references [21] and [19] for further details. The refractive index of bulk material (*N*) was assumed to be 1.33, i.e. equal to that of water. The real part of the refractive index of melanin (*n*) has been determined experimentally to be 1.7 [22]. Mie theory states that the imaginary part of the refractive index is related to the absorption coefficient (*μ_a_*) by [20]:

| $\mu_{\text{a}}=\frac{4\pi n'}{\lambda}.$ | (E) |
| --- | --- |

The absorption coefficient profile of a single melanin granule varies as [23]:

| $\mu_{\text{a}}=c\cdot\lambda^{-3.48}.$ | (F) |
| --- | --- |

A value for *c* = 6.49 · 10^12^ was determined for melanin from RPE cells [23]. By combining Eqs. (E) and (F), we can calculate that (*n’*) ranges from 0.0137 (*λ* = 380 nm) to 0.0035 (*λ* = 700 nm). This provided us with enough information to calculate *g*; and consequently the angular scattering functions of melanin granules. The HG-function only describes the angle-dependent behavior of scattering; the calculation of the scattering cross section *σ*_s_ has to be done by other means. To this end, we calculated the total *σ*_s_ on the basis of Mie scattering as described previously [20, 24], and inserted this as a separate factor into the HG expression.

**C (a). Light absorption by lipofuscin in-vivo**

As stated in section 2.3 of the paper, we accounted for differences in optical absorption by lipofuscin by factoring in the product of the granule concentration and optical path length (*n_g_*· *l*). Paramacular RPE cells have a density of 5000 cells·mm^-2^ [25] and an average cell height of 8.8 μm [15]. Lipofuscin granules constitute 7 - 9% of the cell between age 11-50 and 12% between age 51-70 [10]. Granules are present both in the periphery [25] and surrounding the nucleus [26], and lose their polarized distribution from apical melanin granules with advancing age [15]. With a granule diameter of 1 - 1.2 μm in the adult RPE [27], we can then calculate a concentration of 1.16 – 2.58·10^11^ lipofuscin granules·ml^-1^ (136 – 303 granules per cell). The optical path length in aforementioned in-vitro experiment of oxygen uptake by lipofuscin granules was 332.5 μm (*n*_medium_= 1.33, cuvette path length = 0.25 mm) and the granule concentration was 7.6·10^9^ granules·ml^-1^ [28]. In paramacular RPE cells, the average optical path length varies from 7.8 μm (basal 66% at age 0-30) to 9.1 μm (basal 78% at age 31-50) to 10.4 μm (basal 89% at age 51-80). In patients with Stargardt disease, it is 9.4 μm (basal 80% at all ages).

Values of fundus AF in the posterior pole were based on measurements performed in the presence of a calibrated fluorescence reference to normalize fundus AF intensities for fluctuations in laser power and detector gain [29]. The measured values (‘qAF’) were averaged over 8 specific regions of the posterior pole (see inset in Fig. 6 of the main paper). The resulting values (‘qAF_8_’) were reported, and a best-fit model of the average qAF_8_ for a given age was developed based on a 277 healthy individuals aged 5 - 60 [30]. For healthy individuals, we calculated average qAF_8_ values based on this model for the same age ranges used to calculate (*n_g_*· *l*) as specified above. An average qAF_8_ value for patients with STGD1 was calculated based on values reported previously (Fig. 3 in paper by Burke et al., 2013 [31]), although we decided to only average qAF_8_ values of the 35 patients below the age of 30. We have two reasons for doing so. First, no correlation of qAF_8_ with age was found for patients with STGD1 [31]. Second, for older individuals the presence of atrophic RPE was noted in the 8 regions used for calculating qAF_8_, which may artificially lower their qAF_8_ values [31]. The reported qAF_8_ levels [31] appeared to decline sharply beyond the age of 30.

**C (b). Size of lipofuscin granules of healthy subjects of different ages**

Any age-related differences in the sizes of lipofuscin granules can change the amount of scattering of light passing through a layer with these granules. We therefore analyzed electron microscopy images published previously by Boulton et al. (1990, [32], Fig 1[a-c]) for such age-related differences in granule size.

We manually delineated individual, visually distinguishable, granules in these images using the Freehand-selections tool of ImageJ, and measured the area of each in pixels. By means of an unpaired, two-tailed Student’s t-test with Welch’s correction (i.e. assuming no equal variances), we analyzed the resulting data for differences in the means of three age groups of healthy eyes; 5-29 year old, 30-49 year old, and >50 year old. We used Graphpad Prism ver. 5.03 for this statistical test.

We found no significant differences in the mean lipofuscin granule size between any of the age-groups considered.

1. **Oxygen photoconsumption by lipofuscin**

Rates of oxygen photoconsumption, at ages ≤40 and 41-80 years, were normalized to the incident photon fluxes and to the concentration of lipofuscin granules (7.6·10^9^ granules·ml^-1^) by Rozanowska and co-workers [28]. Therefore, we converted the retinal exposures during AF imaging and exposure to daylight (in mW·cm^-2^) to wavelength-dependent incident photon fluxes (*Φ_p_*) by $\Phi_{\text{p}}(s^{-1}cm^{-2})=H_{r}/E_{\text{photon}}=H_{r}\cdot(\lambda/hc),$ where  *E*_photon_ denotes the photon energy, *h* the Planck constant, and c the speed of light in vacuum (Eq. H). At a normalized *Φ_p_* of 1.61·10^16^ photons·cm^-2^·s^-1^, the fluence rate ranges from 5.7 – 10 mW·cm^-2^, which agrees well with the range at the sample surface reported earlier (0.5 – 12 mW·cm^-2^) [28].

The pH of the solvent used (PBS) and RPE cytoplasm is the same (7.4), although the temperatures differ (*T_in vitro_* ≈ 21°C; *T_in vivo_* ≈ 36.5°C). The maximum oxygen concentration during aforementioned oxygen uptake measurements was 0.26 mM [28]. In the RPE of a light-adapted retina in-vivo this value should be less than 0.123 mM, which is the concentration of oxygen solubilized in pure water at a partial oxygen pressure of about 75 mm Hg [33, 34]. Note that the yield of singlet oxygen increases substantially at oxygen-saturated conditions relative to air-saturated conditions [35].

We propose to calculate oxygen uptake by lipofuscin as in Eq. (G). Here, we correct for differences in the influx of photons *Φ_p_*, media absorption and screening by melanosomes, for the increased optical absorption by lipofuscin in-vivo, and for differences in the concentration of solubilized oxygen relative to that used previously. This is based on the assumption that the rate of oxygen uptake by lipofuscin is proportional to the concentration of solubilized oxygen. We obtain:

|  | ((G) |
| --- | --- |
|  |  |

where

| $\Phi_{p}\text{ (}in\text{ }vivo\text{)}=\left(\frac{H_{r}}{E_{photon}}\right)\cdot\exp\left(-OD_{\text{melanin, }in\text{ }vivo}\right)=H_{r}\cdot\left(\frac{\lambda}{h\cdot c}\right)\cdot\exp\left(-OD_{\text{melanin, }in\text{ }vivo}\right).$ | ((H) |
| --- | --- |

1. **Incorporated parameters**

| **Table S1.** List of parameters used in this study. | | | |
| --- | --- | --- | --- |
| **Parameter** | **Definition** | **Parameter** | **Definition** |
| *H_r_* | Retinal exposure | *H_c_* | Corneal exposure |
| *A*_pupil_ | Pupil area | *A*_retina_ | Retinal area |
| *τ* | Optical transmission through the lens and ocular media | *D_λ_* | Wavelength-dependent optical density of ocular lens and media |
| *d_p_* | Pupil diameter | *f_e_* | Focal length of the eye |
| *α* | Visual angle | *L_s_* | Irradiance of the source |
| Ω | Solid angle | *θ* | Angle subtended by light source |
| *λ* | Wavelength | Φ | Optical power |
|  |  |  |  |
| *μ_a_* | Absorption coefficient | *μ_s_* | Scattering coefficient |
| *μ’_s_* | Reduced/ back scattering coefficient | OD | Optical density |
| *ε* | Molar extinction coefficient | *C* | Concentration (mol·L^-1^) |
| *l* | Optical path length | M | Molarity (mol·L^-1^) |
| *σ_a_* | Absorption cross-section | *σ_s_* | Scattering cross-section |
| *D* | Particle diameter | Q_abs_ | Absorption efficiency |
| *N* | Refractive index (bulk material) | *m* | Complex refractive index (granules) |
| *n* | Real refractive index | *n’* | Imaginary refractive index |
| *g* | Scattering anisotropy factor | *X* | Particle aspect ratio |
| *c* | Scaling factor of melanin absorption coefficient profile | *n_g_* | Granule concentration |
| qAF_8_ | Calibrated fundus AF intensity, average of 8 areas in the posterior pole | *Φ_p_* | Photon flux |
| *E*_photon_ | Photon energy | *h* | Planck constant |
| c | Speed of light in vacuum | *T* | Temperature |

# References

1. Winn B, Whitaker D, Elliott DB, Phillips NJ. Factors affecting light-adapted pupil size in normal human subjects. Invest Ophthalmol Vis Sci. 1994;35: 1132-1137.

2. Illuminance – Recommended light levels. The Engineering Toolbox. 2013 Available from: http://www.engineeringtoolbox.com/light-level-rooms-d_708.html.

3. de Mul FF. Monte-Carlo simulations of light scattering and absorption in turbid media, like tissue, including fluorescence, Raman, laser-Doppler and photo-acoustics. de Mul FF. 2016. Available from: http://www.demul.net/frits/Monte-Carlo.html.

4. de Mul FF, Koelink MH, Kok ML, Harmsma PJ, Greve J, Graaff R, et al. Laser Doppler velocimetry and Monte Carlo simulations on models for blood perfusion in tissue. Appl Opt. 1995;34: 6595-611.

5. de Mul FF. Monte-Carlo simulation of light transport in turbid media. In: Tuchin VV, editor. Handbook of coherent domain optical methods, Biomedical diagnostics, Environment and material science. New York: Kluwer Publishers; 2004. pp. 465-533.

6. Sarna T, Schwartz HM. The physical properties of melanins. In: Nordlund JJ et al. editors. The Pigmentary System: Physiology and Pathophysiology. New York: Oxford University Press; 1988. pp 333-357.

7. Jacques SL. Optical properties of biological tissues: a review. Phys Med Biol. 2013;58: R37-R61.

8. Simon JD, Hong L, Peles DN. Insights into melanosomes and melanin from some interesting spatial and temporal properties. J Phys Chem B. 2008;112: 13201-13217.

9. Zhang QX, Lu RW, Messinger JD, Curcio CA, Guarcello V, Yao XC. In vivo optical coherence tomography of light-driven melanosome translocation in retinal pigment epithelium. Sci Rep. 2013;3: 2644.

10. Feeney-Burns L, Hilderbrand ES, Eldridge S. Aging human RPE: morphometric analysis of macular, equatorial, and peripheral cells. Invest Ophthalmol Vis Sci. 1984;25: 195-200.

11. Weibel ER, Kistler GS, Scherle WF. Practical stereological methods for morphometric cytology. J Cell Biol. 1966;30: 23-38.

12. Dryja TP, O'Neil-Dryja M, Albert DM. Elemental analysis of melanins from bovine hair, iris, choroid, and retinal pigment epithelium. Invest Ophthalmol Vis Sci. 1979;18: 231-236.

13. Sardar DK, Mayo ML, Glickman RD. Optical characterization of melanin. J Biomed Opt. 2001;6: 404-411.

14. Feeney L. Lipofuscin and melanin of human retinal pigment epithelium. Fluorescence, enzyme cytochemical, and ultrastructural studies. Invest Ophthalmol Vis Sci. 1978;17: 583-600.

15. Weiter JJ, Delori FC, Wing GL, Fitch KA. Retinal pigment epithelial lipofuscin and melanin and choroidal melanin in human eyes. Invest Ophthalmol Vis Sci. 1986;27: 145-152.

16. Feeney-Burns L, Burns RP, Gao CL. Age-related macular changes in humans over 90 years old. Am J Ophthalmol. 1990;109: 265-278.

17. Eagle RC, Jr., Lucier AC, Bernardino VB, Jr., Yanoff M. Retinal pigment epithelial abnormalities in fundus flavimaculatus: a light and electron microscopic study. Ophthalmology. 1980;87: 1189-200.

18. Bergougnoux L, Misguich-Ripault J, Firpo JL, Andre J. Monte Carlo calculation of backscattered light intensity by suspension: comparison with experimental data. Appl Opt. 1996;35: 1735-41.

19. Cracknell KP, Farnell DJ, Grierson I. Monte Carlo simulation of latanoprost induced iris darkening. Comput Method Programs Biomed. 2007;87: 93-103.

20. van de Hulst HC. Light scattering by small particles. New York: Dover Publications Inc; 1981.

21. Bohren CF, Huffman DR. Absorption and scattering of light by small particles. New York: John Wiley & Sons Inc.; 1983.

22. Vitkin IA, Woolsey J, Wilson BC, Anderson RR. Optical and thermal characterization of natural (*Sepia officinalis*) melanin. Photochem Photobiol. 1994;59: 455-462.

23. Jacques SL. Melanosome absorption coefficient. Oregon Medical Laser Center. 1998. Available from: http://omlc.org/spectra/melanin/mua.html.

24. Zijp JR, ten Bosch JJ. Pascal program to perform Mie calculations. Opt Engin. 1993;32: 1691-1695.

25. Ach T, Huisingh C, McGwin G, Jr., Messinger JD, Zhang T, Bentley MJ, et al. Quantitative autofluorescence and cell density maps of the human retinal pigment epithelium. Invest Ophthalmol Vis Sci. 2014;55: 4832-4841.

26. Zareba M, Skumatz CM, Sarna TJ, Burke JM. Photic injury to cultured RPE varies among individual cells in proportion to their endogenous lipofuscin content as modulated by their melanosome content. Invest Ophthalmol Vis Sci. 2014;55: 4982-4990.

27. Haralampus-Grynaviski NM, Lamb LE, Clancy CM, Skumatz C, Burke JM, Sarna T, et al. Spectroscopic and morphological studies of human retinal lipofuscin granules. Proc Natl Acad Sci U S A. 2003;100: 3179-3184.

28. Rozanowska M, Pawlak A, Rozanowski B, Skumatz C, Zareba M, Boulton ME, et al. Age-related changes in the photoreactivity of retinal lipofuscin granules: role of chloroform-insoluble components. Invest Ophthalmol Vis Sci. 2004;45: 1052-1060.

29. Delori F, Greenberg JP, Woods RL, Fischer J, Duncker T, Sparrow J, et al. Quantitative measurements of autofluorescence with the scanning laser ophthalmoscope. Invest Ophthalmol Vis Sci. 2011;52: 9379-9390.

30. Greenberg JP, Duncker T, Woods RL, Smith RT, Sparrow JR, Delori FC. Quantitative fundus autofluorescence in healthy eyes. Invest Ophthalmol Vis Sci. 2013;54: 5684-5693.

31. Burke TR, Duncker T, Woods RL, Greenberg JP, Zernant J, Tsang SH, et al. Quantitative fundus autofluorescence in recessive Stargardt disease. Invest Ophthalmol Vis Sci. 2014;55: 2841-2852.

32. Boulton M, Docchio F, Dayhaw-Barker P, Ramponi R, Cubeddu R. Age-related changes in the morphology, absorption and fluorescence of melanosomes and lipofuscin granules of the retinal pigment epithelium. Vision Res. 1990;30: 1291-303.

33. Linsenmeier RA, Yancey CM. Effects of hyperoxia on the oxygen distribution in the intact cat retina. Invest Ophthalmol Vis Sci. 1989;30: 612-618.

34. Linsenmeier RA, Braun RD. Oxygen distribution and consumption in the cat retina during normoxia and hypoxemia. J Gen Physiol. 1992;99: 177-197.

35. Rozanowska M, Wessels J, Boulton M, Burke JM, Rodgers MA, Truscott TG, et al. Blue light-induced singlet oxygen generation by retinal lipofuscin in non-polar media. Free Radic Biol Med. 1998;24: 1107-1112.
